# Binary Neural Networks in FPGAs: Architectures, Tool Flows and Hardware Comparisons

**DOI:** 10.3390/s23229254

**Published:** 2023-11-17

**Authors:** Yuanxin Su, Kah Phooi Seng, Li Minn Ang, Jeremy Smith

**Affiliations:** 1 School of AI and Advanced Computing, Xi’an Jiaotong Liverpool University, Suzhou 215000, China; yuanxin.su22@student.xjtlu.edu.cn; 2 Department of Electrical Engineering and Electronics, University of Liverpool, Liverpool L69 3GJ, UK; j.s.smith@liverpool.ac.uk; 3 School of Computer Science, Queensland University of Technology, Brisbane City, QLD 4000, Australia; lang@usc.edu.au; 4 School of Science Technology and Engineering, University of the Sunshine Coast, Sippy Downs, QLD 4556, Australia

**Keywords:** field-programmable gate array (FPGA), binary neural network (BNN), latency reduction, computational modeling

## Abstract

Binary neural networks (BNNs) are variations of artificial/deep neural network (ANN/DNN) architectures that constrain the real values of weights to the binary set of numbers {−1,1}. By using binary values, BNNs can convert matrix multiplications into bitwise operations, which accelerates both training and inference and reduces hardware complexity and model sizes for implementation. Compared to traditional deep learning architectures, BNNs are a good choice for implementation in resource-constrained devices like FPGAs and ASICs. However, BNNs have the disadvantage of reduced performance and accuracy because of the tradeoff due to binarization. Over the years, this has attracted the attention of the research community to overcome the performance gap of BNNs, and several architectures have been proposed. In this paper, we provide a comprehensive review of BNNs for implementation in FPGA hardware. The survey covers different aspects, such as BNN architectures and variants, design and tool flows for FPGAs, and various applications for BNNs. The final part of the paper gives some benchmark works and design tools for implementing BNNs in FPGAs based on established datasets used by the research community.

## 1. Introduction

In recent years, convolutional neural networks (CNNs) have showcased remarkable performance across various domains, including image classification [1,2], object recognition [3,4], speech emotion recognition [5,6] and the classification of noise from non-stationary signals [7]. Typically, CNNs utilize 32-bit float-pointing values for training and inference. This requires devices with high computation capability and a substantial storage space. This requirement makes deep learning techniques dependent on devices with high computation performance like GPUs. However, in many real-world applications, we may need to implement deep learning neural networks in devices with limited resources. For example, embedded systems based on field-programmable gate arrays (FPGAs) only have a few thousands of units for vector computation, which is insufficient for dealing with common deep learning models with millions of parameters. Therefore, many researchers have proposed different techniques to compress the memory footprint and computation requirements of neural networks [8].

Binary neural networks (BNNs) can be considered as extreme cases of quantization. The idea of BNNs began with BinaryConnect [9], proposed by Courbariaux et al. They developed a deep neural network which represents weights by the binary values {−1,1} in forward propagation and updates the binary weights from a gradient of real values during training. Even though BinaryConnect applies binary weights, the activation function still uses full-precision representation. To improve BinaryConnect, Courbariaux et al. also proposed a modification that uses binary values to represent both the weights and the activation. It is defined as the first binary neural network [10]. Additionally, the work achieved a 32-times compression ratio on parameters and a speed-up of approximately 7 times on the MNIST, CIFAR10 and SVHN datasets. However, the experimental results showed that the BNN in [10] was not suitable for big datasets (e.g., ImageNet [11]). Therefore, XNOR-Net [12] was proposed. The authors introduced a scaling factor to reduce quantization error and increase accuracy. They also used XNOR-bitcount to replace the convolution operation, which significantly reduced the matrix computation cost. Finally, they reduced the memory footprint by a factor of 32, and the system was 58 times faster at performing the convolution operation than the full-precision version. These two pioneering works, BinaryConnect and XNOR-Net, demonstrated the capacity of binary neural networks. However, BinaryConnect must lose a lot of information because of the decreasing representation ability caused by binarized values. Even though XNOR-Net alleviates the problem to some extent with statistical scaling factors, this is accompanied by a high computational overhead due to the statistical features.

The authors of DoReFa-Net [13] and Bi-Real Net [14] recognized the problem and proposed a solution. DoReFa-Net used a static scaling factor to retain more information while avoiding the burden of real-time computation of statistical features during inference. And the authors of Bi-Real Net focused on minimizing error during backward propagation by replacing the sign function with a specially designed approximation. At the same time, Bi-Real Net also introduced a full-precision shortcut to enhance the information of the feature maps. Then, XNOR-Net++ not only proposed a way of merging the activation and weight scaling factor as a single learnable parameter, but also explored the influence of scaling factors with different shapes. Except for the scaling factors, Bethge, J et al. thought that a good network structure is the key to BNNs and summarized the design philosophy of the BNN structure to define what kind of structure is a good structure for BNNs [15]. On the other hand, ReActNet [16], Adabin [17] and DyBNN [18] worked on improving the performance of BNN by setting the shifting or threshold parameters of the activation function as the learnable parameters to change the properties of the activation function. But because the parameters are learnable, if the parameter cannot match with a suitable value during the training process, the method will cause significant information loss instead. IE-Net improved this problem without adding much computational burden by deploying multiple activation functions simultaneously and sharing the convolutional layer [19]. IR-Net [20] differs from the usual BNN measure of quantization error by introducing information entropy to measure quantization error from a novel perspective. And IR-Net also proposed a hardware-friendly scaling factor. The Binarized Ghost Module (BGM) [21] and RB-Net [22] enhanced the representation capacity of feature maps through reshaping and concatenating operations.

Based on our study of the literature, there have been a number of BNN-related reviews, ranging from conceptual summaries [8,23,24,25] to technical summaries related to various domains, such as agriculture [26], medicine [27,28,29], large-scale image retrieval [30], human action recognition [31,32], etc. This means significant research has been devoted to BNNs, applying them in many areas, including object recognition [31,33,34,35], semantic segmentation [36,37] and point-cloud tasks [38]. Compared to other BNN-related reviews and earlier works, this review contributes the following:

(1) It covers a wide spectrum of AI-enabling technologies for BNNs by summarizing more existing works published up until early 2023.
(2) BNN architectures, models and principles within the main categories of techniques are analyzed and discussed.
(3) It covers the mainstream tool flows for machine learning to FPGAs. It introduces the key design and principle of each tool and also summarizes the workflow of each tool flow.
(4) Identifying current challenges and future directions for BNNs in resource-limited devices, the paper also offers some comparisons of BNN architectures and benchmarking results to give insights into FPGA implementation.

Regarding the paper’s structure, Section 2 presents the tool flows and their key designs for hardware, particularly FPGA implementation. The further development of BNNs is discussed in Section 3. Section 4 gives a comparison of BNN architectures and benchmarking results for FPGA implementation using established datasets. Section 5 discusses future work and challenges for BNNs, and Section 6 gives some concluding remarks.

## 2. Survey of Tool Flows for Machine Learning (ML) to FPGAs

This section describes the various design tools and workflows which have been proposed for machine learning using FPGA implementations. The following tool flows are covered: (1) HLS4ML [39], (2) FINN [40,41], and (3) TVM/VTA [42].

### 2.1. Resources on Boards

Before focusing on tool flows, there are some resources (DSPs, LUTs and BRAM) on boards which need to be introduced. A digital signal processing (DSP) block is composed of a high-performance hardware multiplier plus some accumulators and registers, multiplexers and other resources. DSPs provide FPGAs with the ability to perform powerful digital signal processing operations by combining these high-performance resources in close proximity. However, the number of DSP blocks is typically limited in FPGAs.

A lookup table (LUT) is essentially an RAM, where the user describes a logic circuit through schematic or HDL language; the FPGA development software (Xilinx Vivado 2018.1) will automatically calculate all the possible results of the logic circuit, listed in the form of a truth table, and write the truth table (i.e., the output logic corresponding to the inputs) into the RAM in advance, so that each input signal for the logic operation is the same as inputting an address to look up the table to find out what the address corresponds to and then output it. The table is then written into RAM, so that each signal input for the logic operation is equivalent to inputting an address, looking up the table, finding out what the address corresponds to and outputting it.

Block RAM (BRAM) is one of the most important storage resources in FPGAs. BRAM is characterized by high speed, reliability and low power consumption, and is widely used to store large amounts of data and programs. In FPGA design, we can configure BRAM through IP, Verilog code, etc., to adapt it to different application scenarios.

### 2.2. HLS4ML

**Figure 1:**
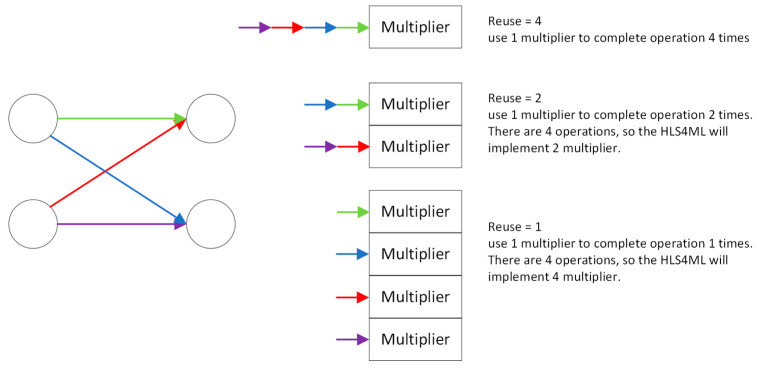
Reuse factor.

HLS4ML is an automatic tool for deploying neural networks in FPGAs with ultra-low latency and low power [39]. HLS4ML has two important design features. One of those is parallelism, where the core of the convolutional layer can be regarded as matrix–vector multiplication. The team of HLS4ML introduced a factor called the reuse factor. The reuse factor is a parameter for control of the parallel or streaming model implementation with varying degrees of pipelining. The effect of the reuse factor is shown in Figure 1. In the Figure 1, the different color arrows mean different datapaths with different multipliers.

Another important design feature is quantization-aware training (QAT). As discussed above, a neural network with full precision will be difficult to implement in devices with limited resources. The team of HLS4ML introduced QKeras [43] to quantize the model to achieve the best performance at low precision and benefit automatically during inference, with HLS4ML parsing of QKeras models.

**Figure 2:**
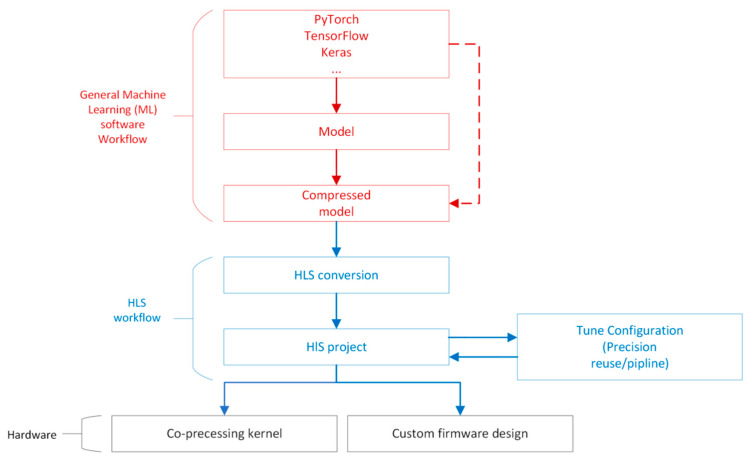
HLS4ML workflow overview.

The workflow of HLS4ML can be summarized as follows and the overview is shown as Figure 2:

(1) Training the model and then undertaking compression (pruning and quantization) using a deep learning framework (Keras, TensorFlow or pytorch).
(2) The HLS4ML package will convert the model to an HLS project and generate an Intellectual Property (IP) module.
(3) Each layer and activation can be implemented as a separate configurable module which contains computational modeling of each layer. Configurations include clock period, IO type, bitwidth, reuse factor, and so on.
(4) In Vivado, importing the IP generated by HLS4ML and then connecting to the PS (Processing System) and analyzing the resource cost, e.g., DSP, LUT, BRAM, and so on.
(5) Synthesis and deployment in an FPGA.

In [44], the team that developed HLS4ML proved the capability of HLS4ML in terms of binary or ternary neural networks. In the experiment, they implemented four layers of fully connected neural networks on LHC [45] and MNIST [46]. The results demonstrated that the implemented binary or ternary versions of the models have similar performance to high-precision models, while the utilization of DSPs is nearly non-existent. Compared to the baseline model, the model with binary values only needed half the BRAM. As HLS4ML was refined, the researchers could apply quantization neural networks to more areas. Gheilmetti et al. [47] quantized ENet [48] from full-precision to 8-bit and 4-bit fixed-point data types and even binary data types. The results demonstrated that the quantized ENet gains 4.9 ms latency per image and uses less than 30 percent of board resources in the Xilinx ZCU102 evaluation board.

### 2.3. FINN

FINN [40] is another bridge between FPGAs and neural networks. The tool was published by Xilinx, so it supports many different Xilinx FPGA boards. It specifically targets quantized neural networks (QNNs), with emphasis on generating data-flow-style architectures customized for each network. In addition, Xilinx also developed Brevitas, a Pytorch-based quantization-aware training toolkit, for FINN. To achieve high flexibility, Xilinx separated out a library called finn-hlslib, which can highly customize configurable C++ template parameters, such as the data type of the input, the weight and output, and the mapping of different FPGA resources, like LUTs or DSPs and LUTRAM or RAM.

Generally, there are two possible solutions for implementing a neural network in an FPGA: (a) the traditional RTL design by VHDL and (b) the HLS design flow using C++. The FINN team chose solution (b) to develop their tool flow because the benefits of using HLS include design productivity, portability to several different platforms, built-in optimization for data flow and automatic flow control. Nevertheless, these benefits come at the cost of a degree of resource overhead. To be more specific, several experiments [41] show that HLS implementation will increase resource overhead by 45% compared to VHDL implementation using the method provided by Thomas B. Preußer [49].

**Figure 3:**
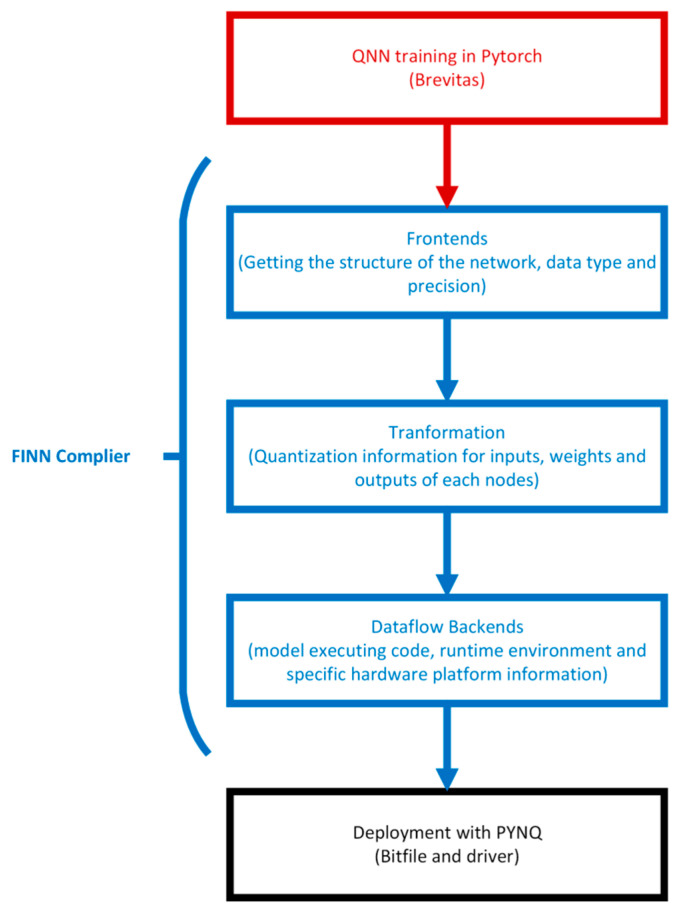
Overview of FINN workflow.

As shown in Figure 3, the first step of FINN is training a quantized neural network in PyTorch with Brevitas; then, the model will import into the FINN compiler. The processes of the FINN compiler can be split into front-end and back-end processes. In the front end, FINN converts the QNN trained by the framework to FINN intermediate representation (IR) to obtain the structure of the network, including data type and precision.

The intermediate representation (IR) contains quantization information for the inputs, weights and outputs of each node. This information is essential for quantization-aware optimization and the mapping of back-end optimization for quantized computing.

The back end is a deployment package which is created using an IR diagram and back-end-specific information. The deployment package includes parameters for the QNN model, back-end-specific code for executing the model, the runtime environment, executable hardware designs for targeting the data streams and the multilayer offloading architecture, and a selection of predefined platforms.

**Figure 4:**
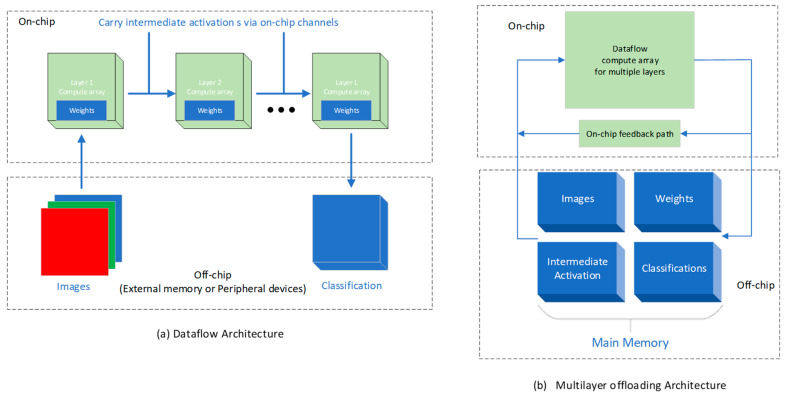
Data-flow architectures [41].

FINN also supports two different accelerator architectures, data flow and multilayer, for dealing with various hardware resources [41]. In the data-flow architecture, FINN will be customized for a specific neural network topology, with different accuracy for the activations and weights for each layer. Each layer instantiates a computational engine. As soon as the previous engine starts producing output, the engine starts computing, thus introducing another kind of concurrency between the layers. On the other hand, a multilayer architecture is equivalent to taking the concatenation of computational resources in a data-flow architecture suitable for large networks under limited resource constraints. In conclusion, the cost of a data-flow architecture is the sum of all the implemented layers, while the cost of a multilayer architecture is defined by the maximum value of the layer. Obviously, the former has lower latency but a higher resource overhead. A diagram of the two architectures is shown as Figure 4.

**Figure 5:**
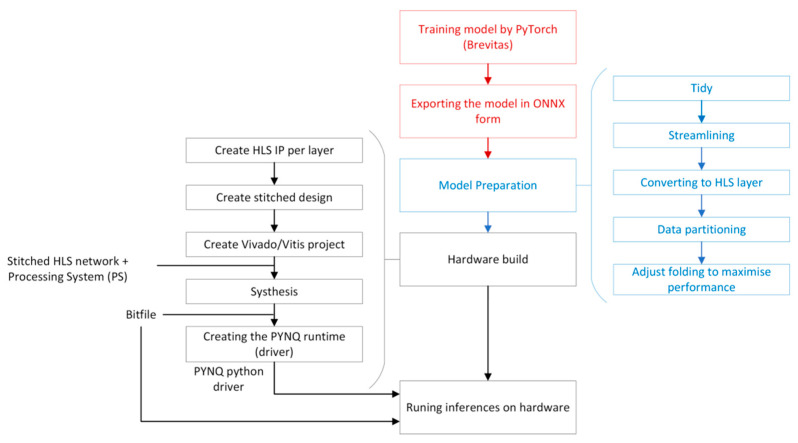
FINN workflow.

After the general introduction of the FINN workflow, it is essential to understand the actual specific workflow in practice. Figure 5 shows the specific FINN workflow, from software to hardware runtime. The red blocks indicate the general software steps for training a model by PyTorch using the library Brevitas, which is specialized for QNNs. Once there is a trained QNN in ONNX format, the model can be imported into the FINN compiler for model preparation.

In model preparation, FINN provides plenty of functions to make a model more readable and simplify the computational process. For example, after the Tidy process, each layer of the model will be given a unique name according to operation, mul_0 or maxpool_0, rather than an initialized name, layer 1, 2, 3, and so on. This process improves readability while providing recognizable keywords for all the functions that follow, and all functions can be automatically simplified by keywords. Except for the unique name, the process also labels all of the information about tensor shape and data type; so, for BNNs, FINN can transform multiplication in a convolutional layer that has binarized weights and inputs into a XNOR-popcount operation automatically.

In streamlining, all the scaling layers in front of a quantization layer will collapse into a linear transform. And then the linear transform can be fully merged into the quantization by updating its threshold, which saves storage and computing overhead [50]. Next, the FINN compiler will convert nodes to custom nodes corresponding to the FINN-hlslib function. In other words, the compiler will map the layers to predefined HLS layers in the library to FINN-hlslib. At the same time, data partitioning can separate out the HLS layers and non-HLS layers in the model, where non-HLS layers are some layers that the PS can deal with, such as pre-processing (ToTensor and normalization) and post-processing (Top-K algorithm). Finally, folding processing aims to adjust the parameters for controlling the parallelism of computation that are inserted in the process of conversion to HLS layers.

**Figure 6:**
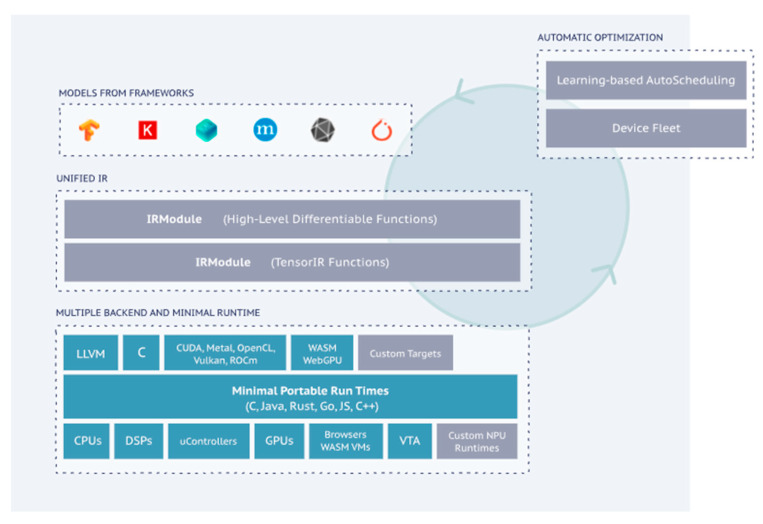
Overview of TVM framework [51].

All the operations in the black box of Figure 6 will be completed automatically by a hardware function provided by FINN. Users only need to specify the hardware platform or the Xilinx FPGA part; FINN can automatically generate the corresponding Vivado project through FPGA synthesis tools (Vitis HLS and Vivado). In the Vivado project, users can add a customized design in the block design and also analyze the hardware resources. Furthermore, FINN also generates PYNQ drivers for implementation.

### 2.4. TVM/VTA

**Figure 7:**
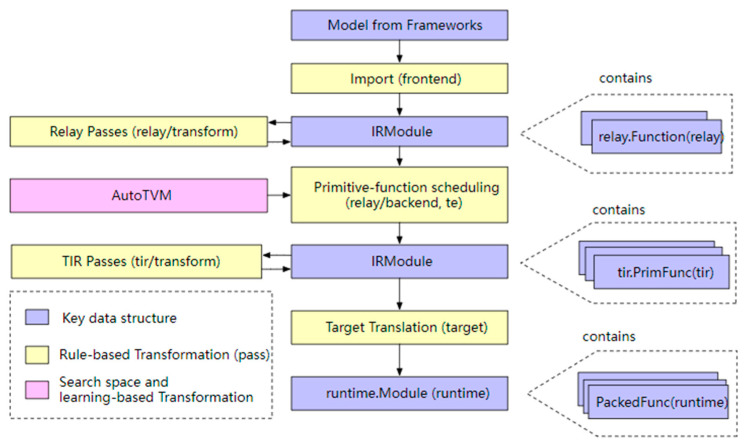
Overview of TVM workflow [51].

TVM [42] is an open-source, end-to-end deep learning model compilation framework for optimizing the inference operation speed of deep learning models in arbitrary target environments, such as CPUs, GPUs, ARMs, etc. Common applications include scenarios that require compatibility with all mainstream models as input and the generation of optimized deployment models for any type of target hardware; scenarios that have strict requirements on the inference latency, throughput and other performance indicators of the deployed models; and scenarios that require custom model operators, self-developed target hardware and custom model optimization processes. The workflow can be summarized as follows: the models derived from common deep learning frameworks (Tensorflow, Pytorch, MXNet, etc.) are used as inputs to the TVM framework, and after a series of graph optimization operations within the framework and automatic optimization operations at the algorithm level, they are eventually transformed into deployment models, like CPUs, GPUs and ARMs, for the target runtime. The optimized models theoretically maximize the utilization of the target hardware’s resources in order to minimize the model’s inference latency. An overview of the TVM framework is shown below:

The TVM compiler is the main functional component of the TVM, which is responsible for optimizing and compiling the deep learning model into a code that can run inference tasks on the target device. The overall compilation process is shown in Figure 7.

In the figure, the blue operations represent the data structures involved in the TVM compilation process, the yellow operations represent the algorithms that process these data structures and the pink AutoTVM is a special algorithm that assists the Schedule function in selecting parameters. The data structure of the entire TVM compilation stack consists of:

(1) A model from a framework: a model exported from PyTorch, MXNet, etc.
(2) The IR Module (Relay): the intermediate representation (IR) in the level of the TVM graph.
(3) The tensor-level IR (TIR) for TVM, which contains specific scheduling details (loop nesting, parallelism, instruction sets, etc.) for each operator corresponding to the network layer.
(4) The Runtime Module is the lowest-level IR of the TVM compilation stack, directly interfacing with the runtime to run on the target device.

The Versatile Tensor Accelerator (VTA) [52] is a small FPGA-based acceleration back end developed by the TVM compilation stack. Running end-to-end computational tasks on the VTA requires a complete software stack that maps high-level models to the VTA programmable interface. The first step of the conversion process is computation graph-level optimization. In this step, the TVM first receives various deep learning models, such as PyTorch, TensorFlow, MxNet, etc., and converts them into a Relay IR. In the Relay layer, computation graph-level optimization is performed, such as constant folding, dead-code elimination and operator fusion. After that, computation graph wrapping is needed because graph representation and optimization are independent of hardware platforms. The computation graph needs to be wrapped in a hardware-platform-specific format. The following step is the optimization of operators, which defines the algorithmic steps that need to be performed to accomplish the operation, while scheduling specifies how the computation is to be performed, e.g., how the data are to be loaded and stored and what optimizations are to be used, such as loop chunking, loop unrolling, multithreading, etc. Finally, the TVM IR, also called the TIR, is generated after the operator-level optimization, which is hardware-dependent and in which the API provided by the VTA is embedded. After generating the VTA instructions, a run signal is written to the Programmable Logic (PL) of the FPGA device, and the HLS accelerator portion of the VTA starts to perform the computation in a task-level parallel manner, which ultimately realizes the forward inference of the entire neural network.

### 2.5. Optimization

These tool chains also have their own special optimizations for different types of neural networks. Although some of these optimizations are not specific to binary neural networks, binary neural networks can still benefit from them. This is because the goal of all these tool chains is to achieve the lowest latency and the best possible resource utilization. More importantly, most of the existing tool chains do not have enough generalization ability for FPGAs. Most tool chains are well optimized for specific FPGA platforms, so whenever we want to deploy BNNs or QNNs on different FPGA platforms in large quantities, we need to repeat the process of platform-specific optimization, which is very cumbersome and inefficient. However, the three tool chains we discussed above are adapted for a large portion of FPAG platforms, and their optimization methods are very generalizable.

#### 2.5.1. HLS4ML (Optimization for Arbitrary Precision Neural Networks)

The special design of HLS4ML is that it is optimized for both the front end and back end. The optimizations in HLS4ML consist of three main aspects:

(1) Compression: Inspired by [53,54], L1 regularization was added as an additional penalty term to the loss function, L, to obtain a sparse model. L1 regularization has been proven to be a powerful way to generate a sparse weight matrix, which can, in turn, be used for feature selection [55]. The loss function can be expressed as:
  (1) $$L_{\lambda }(W)=L(W)+\lambda ||W|{|}_{1}$$
  where L(W) is the original loss function, the term after the plus sign is L1 regularization and λ is the regularization factor. The authors’ experiments indicate that after seven iterations, in a three-hidden-layer model, they achieved a compression rate of nearly 70%.

(2) Quantization: Optimization in terms of quantization is not specialized for BNNs, but it is worth mentioning. HLS4ML adopts fixed-point arithmetic to achieve lower resource costs and latency than floating-point arithmetic.
(3) Parallelization: As described in Section 2.2, the reuse factor is a key optimization for back-end hardware that affects the parallelism of data flow in hardware. Users can choose the corresponding degree of parallelism based on the number of DSPs their platform has in order to achieve the shortest latency and higher resource utilization.

#### 2.5.2. FINN (Special Optimization for BNNs)

FINN differs from HLS4ML and TVM/VTA in that it has its own special optimizations for binary neural networks. These include the following specific operators:

(1) XNOR and popcount replace the binary dot product and summation to avoid signed arithmetic. According to experimental results that were implemented by Vivado HLS, compared with signed-accumulate, popcount-accumulate only required nearly half the number of LUTs and FFs.
(2) Converging batch normalization [56] and activation as threshold. Normally, a BNN will insert batch normalization between convolutional or fully connected layers and sign functions. A special threshold activation was designed that allowed the computation of activation using unsigned inputs and avoided batch normalization, which requires the utilization of large amounts of hardware resources during inferences. Experiments indicate that using a 16-bit dot product as input, regular batch normalization with sign activation needs 2 DSPs, 55 FFs and 40 LUTs, but the threshold activation only requires 6 LUTs.
(3) Boolean OR for max pooling. Regularly, the pooling layer will perform before batch normalization and the activation function, which means that the pooling layer will have to deal with non-binarized values. FINN shows that the same outputs can be achieved by max pooling that is performed after activation functions without retrain networks. It further optimized the utilization of hardware resources during inference because max pooling is only performed with binarized values.

**Figure 8:**
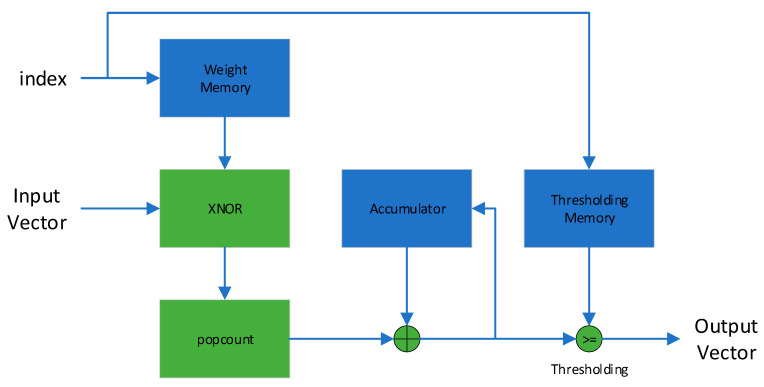
MVTU data path.

Except for the optimizations, FINN also proposed a specially designed computational core for an accelerator called the Matrix–Vector–Threshold Unit (MVTU). In FINN’s implementation, the computation operations in the BNN are regarded as matrix–vector operations with thresholding. The MVTU data path is shown in Figure 8. First, the MVTU computes the dot product of the input vector and one of the rows of the weight matrix. The result of the dot product is then compared with the thresholding value to produce a single-bit value. The dot product consists of two parts, XNOR and popcount, corresponding to the first optimization mentioned above.

#### 2.5.3. TVM/VTA (An Automatic Tool Chain for Various Platforms)

In terms of optimization approach, TVM/VTA differs from traditional tool chains in that it aims to make automatically or semi-automatically generated code as good as handwritten code. Therefore, for TVM/VTA, its optimization is more of a selection process rather than a design process.

For hardware optimization, TVM/VTA provides hundreds to thousands of candidate designs automatically based on the configuration options of users, such as PLL frequency and the degree of hardware pipelining. TVM/VTA then uses a simple FPGA resource model to exclude infeasible solutions. The remaining solutions go through a compilation, layout and routing process, and a few of the best feasible solutions are selected based on the FPGA’s resources, data type and batch size. The rest are excluded due to lower peak performance or because they have layout, routing or timing closure faults. The final set of designs is tuned automatically by a learning-based framework [57] to generate a workload performance profile. Finally, users can choose one solution from the available options.

In the front end, TVM/VTA can also explore the best schedule for deep learning operators. TVM uses the XGBoost [58] search algorithm to automatically achieve the best schedule that maximizes performance using an automated scheduling library based on combinations of operators, tensor shapes and hardware parameterization.

In the optimization case, TVM/VTA achieved a tenfold reduction in latency for Resnet-18 with W8A8 after 4000 optimization iterations.

## 3. Survey of BNN Architectures

This section reviews binary neural networks which represent benchmark results in different stages of training binary networks. Table 1 summarizes all the benchmark results of binary neural networks. 

### 3.1. Binarized Neural Networks (BNNs)

In early binary neural networks (BNNs), the binarization of weights and activation functions simply quantized values from float-point to 1-bit values through a fixed binarization function with down-quantization by taking the signs of weights and inputs. The binarization function (sign function) is defined as:

(2) $$sign(x)=\left\{\begin{array}{ll} -1, & if\;x\leq 0\\ 1, & if\;x>0 \end{array}\right.$$

BinaryConnect [9], proposed by Courbariaux et al., is a pioneer in binary neural network research. It transforms full-precision weights into 1-bit weights. The forward propagation of BinaryConnect uses a stochastic method rather than a sign function to quantize the weights, which reduces the quantization error:

(3) $$W_{b}=\left\{\begin{array}{ll} -1, & with\;probability\;\rho \leq \sigma (\omega ).\\ 1, & with\;probability\;1-\rho . \end{array}\right.$$

where $W_{b}$ denotes binarized weights and ρ is a hard sigmoid function:

(4) $$\sigma (x)=clip(\frac{x+1}{2},0,1)=max(0,min(1,\frac{x+1}{2}))$$

After that, Courbariaux et al. improved their work [10]. In addition to introducing the Straight-Through Estimator (STE) to avoid the problem that the derivative of the sign function is almost zero everywhere, they also devised XNOR-Bitcount and shift-based batch normalization to accelerate the inference of networks. The experiments proved that their method is very powerful in terms of acceleration and training. The results show that their method is 7 times faster in terms of runtime and requires 32 times less memory and memory access.

### 3.2. XNOR-Net

Even though BNNs save computational resources and storage requirements, binarization inevitably causes loss of information, which reduces the accuracy of the networks in varying tasks. Reducing the quantization error is a powerful way to avoid loss of accuracy, which has been proved in the past years. The quantization error in BWN [12] is defined as:

(5) $$J(W_{b},\alpha )=||W-W_{b}|{|}^{2}$$

where J(·) is the loss function and α is the scaling factor, whose optimal value can be calculated by:

(6) $$\alpha^{*}=\frac{1}{n}\|W{\|}_{L1}$$

where n is the number of input pixels and L1 means the L1 norm.

To further minimize the quantization error, XNOR-Net [12] was also proposed by Rastegari et al. XNOR-Net is an extended version of BWM, which also binarizes inputs using the sign activation function with scaling factors. The quantization rule is similar to the quantization rule for weights in BWM:

(7) $$\beta^{*}=\frac{1}{n}\|X{\|}_{L1}$$

where X denotes the inputs of the activation function.

For a given L-layer convolutional neural network, the weights and inputs are denoted as $W\in R^{o\times c\times w\times h}$ and $X\in R^{c\times w_{in}\times h_{in}}$, where w and h are the width and height of the kernels, c is the number of input channels, o is the number of output channels, and $w_{in}$ and $h_{in}$ represent the size (the width and height) of the inputs. The binary convolution operation with scaling factors will become:

(8) $$X*W\approx (\alpha^{*}sign(X)⊛\beta^{*}sign(W))=\alpha^{*}\beta^{*}(sign(X)⊛sign(W))$$

where ⊛ denotes the XNOR bitcount operation.

Except for the quantization method, XNOR-Net also improves training by replacing the pooling layer after the dense layer or convolutional layer, which can avoid the inputs of the pooling layer, which are binarized values. While the introduction of a scaling factor reduces loss of information during the quantization process, the computation of the scaling factor is quite demanding. It has to calculate the L1 norm in every dot product layer and compute the L1 norms of activation inputs in real time in both training and inference, which is quite costly in terms of computing resources.

### 3.3. DoReFa-Net

The shortcomings of the XNOR-Net approach to computing the gain term were pointed out by Zhou et al. DoReFa-Net [13] does not use the L1 norm of activations and weights to dynamically compute the gain term. Instead, the gain term is based only on the weights of the network. Since the weights and gain terms do not change after training, efficient inference can be achieved.

The authors of [13] also proposed a generalized quantization method which was designed to quantize weight, actication and gradient at different bit widths. Also, since the gradient is quantized, there is also a speed-up effect in the backpropagation process. Even though the method proposed in DoReFa-Net is not specialized to binarize neural networks, there are also experimental results for binary neural networks.

### 3.4. Bi-Real-Net

Bi-Real-Net [14] presents a series of enhancements and improvements to address the shortcomings of XNOR-Net, specifically including the introduction of shortcut connections in the form of one layer per block, the use of a quadratic function to fit the sign operation of real activations, the introduction of the magnitude of real weights when updating the real weights and the use of a clip function instead of ReLU to train the pre-trained model.

**Figure 9:**
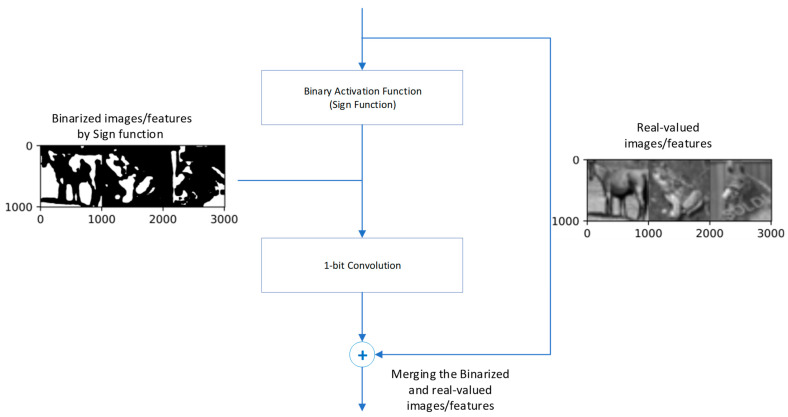
Bi-Real block.

Firstly, the network structure realizes the shortcut connection in the form of one layer per block, i.e., the real-number result output from the current 1-bit convolution or BN is directly added to the real-number result output from the next 1-bit convolution or BN, which increases the network’s numerical representation range (value range). The shortcut structure is shown in Figure 9.

Since the sign function is not differentiable (the derivative is the unit impulse response), it is necessary to design a differentiable function to approximate the sign function. Nevertheless, the approximation of the sign function will cause a mismatch between the approximated gradient and the gradient of the real value in backpropagation, so XNOR-Net uses a clip function as an approximation of the sign function, which suffers from large errors. Zhou et al. [14] design a specialized sign function called the ApproxSign function to get close to the sign function:

(9) $$ApproxSign(x)=\left\{\begin{array}{ll} -1, & if\;x<-1\\ 2x+x^{2}, & if\;-1\leq x<0\\ 2x-x^{2}, & if\;0\leq x<1\\ 1, & otherwise \end{array}\right.$$

(10) $$\frac{\partial ApproxSign(x)}{\partial x}=\left\{\begin{array}{ll} 2+2x, & if\;-1\leq x<0\\ 2-2x, & if\;0\leq x<1\\ 0, & otherwise \end{array}\right.$$

**Figure 10:**
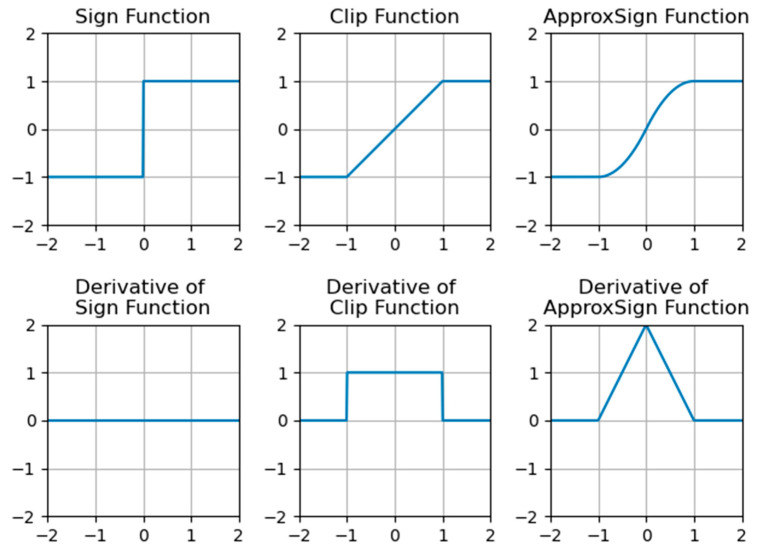
Functions and derivatives.

ApproxSign, as a direct approximation of the sign function, can further reduce the gradient error and enhance model performance. In addition, the curve of the derivative of ApproxSign is triangular in shape, which is more suitable for modeling unit impulse signals. The sign function, the clip function, the ApproxSign function and their derivatives are shown in Figure 10.

### 3.5. XNOR-Net++

Bulat et al. [61] set the scaling factor as a learnable parameter inside the model instead of calculating the statistical feature. XNOR-Net++ [61] reformulates Equation (8) as:

(11) X * W≈(sign(X)⊛sign(W))⊙Γ

where Γ denotes the learnable scaling factor. XNOR-Net++ proposes four ways to compute and compare performance. The scaling factor with the best result is constructed as:

(12) $$\Gamma =\alpha ⊗\beta ⊗\gamma ,\;\alpha \in R^{C_{o}ut},\beta \in R^{h_{out}},\gamma \in R^{w_{out}}$$

where α, β and γ are the rank-1 factor over the output channel, the output height and the output width (C, H and W), respectively.

### 3.6. BinaryDenseNet

Different from other researchers, Bethge, J et. al focus on optimal structure design for binary neural networks [15]. Their research indicates that if binarization is performed on layers that are not shortcut connected, then it will result in an irrecoverable loss of information. So, for BNNs, the first convolutional layer, the last fully connected layer and the downsampling layer should be kept at full precision to avoid further information loss. They conducted experiments on ResNet-18 for the CIFAR-10 dataset. The results show that accuracy improved by 0.4% to 1.1%, but the size of the network increased from 1.39 MB to 2.03 MB.

**Figure 11:**
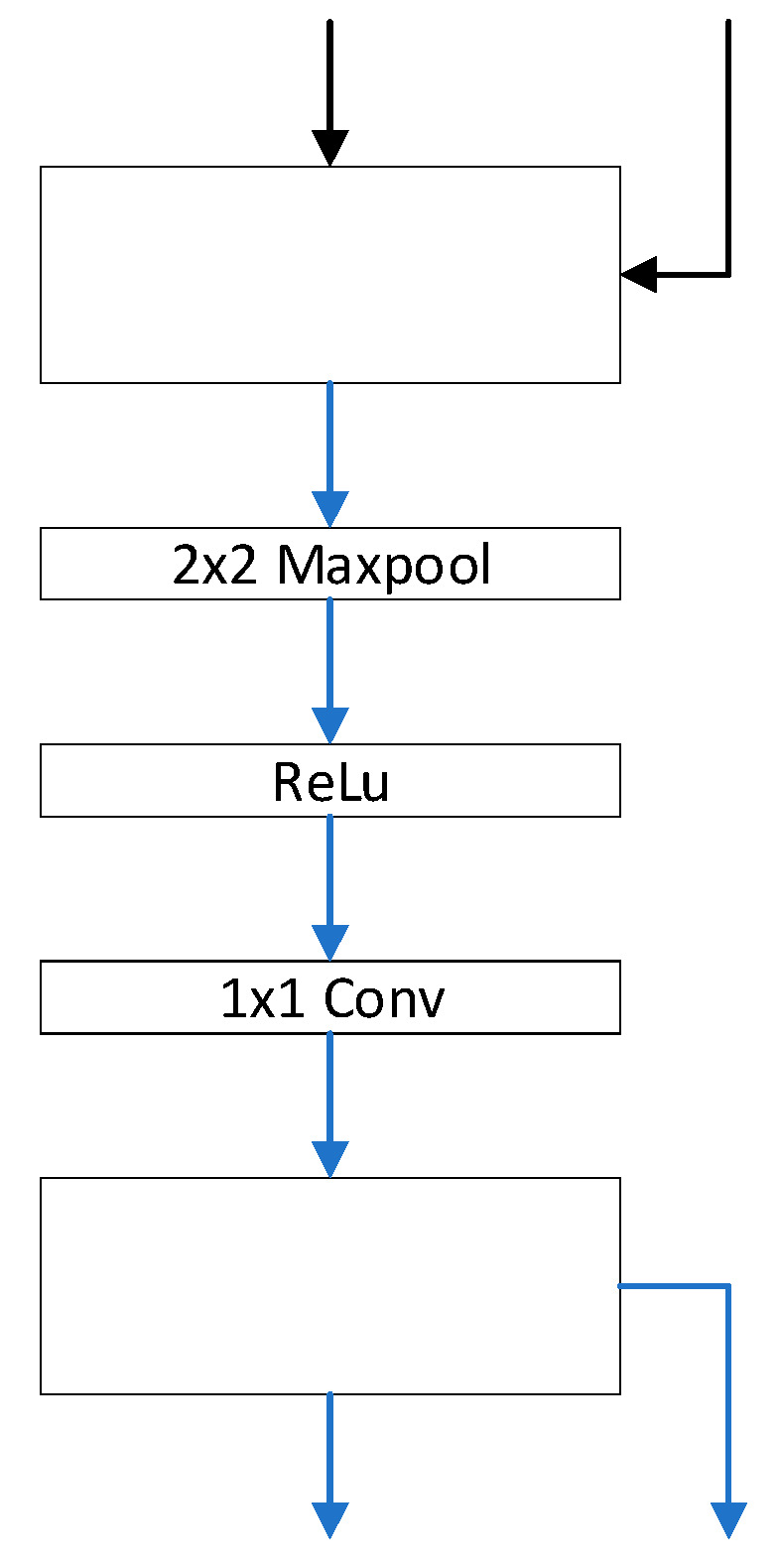
BinaryDenseNet.

In this paper, the authors also summarized several guidelines for the design of BNN structures:

(1) The design philosophy of the BNN structure should be based on maximizing information retention.
(2) Compact network structures may not be suitable for BNNs because compact neural network structures are designed to reduce redundancy, whereas BNNs aim to increase the transfer of information.
(3) A bottleneck structure [59] should be avoided as much as possible. A bottleneck structure first decreases the number of channels and then increases them, which may lead to irreversible information loss in BNNs.
(4) The downsampling layer should maintain full precision.
(5) The shortcut structure preserves information and is friendly to BNNs.
(6) The order of operations to change the shortcut between blocks is Maxpool-ReLU-1x1Conv. The structure presents as Figure 11.

### 3.7. ReActNet

**Figure 12:**
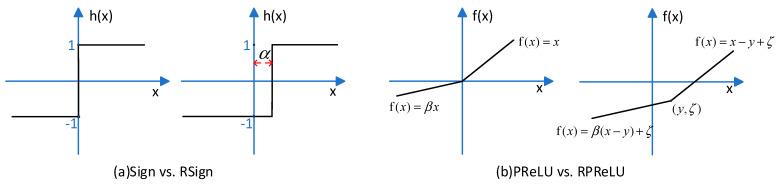
RSign and RPReLU.

Actually, the quantization methods discussed previously can also be regarded as traditional activation functions multiplied by a factor to represent more information. On the other hand, ReActNet [16] changed the distribution of a traditional activation function to dramatically improve accuracy. Liu et al. [16] proposed RSign and RPReLU to replace Sign and PReLU [1]. The function is shown as Figure 12.

Both functions are channel-wise, which means that each channel has its own parameters. The method modifies the means and standard deviations of numerical distributions by simple linear transformations. All of the parameters in RSign and RPReLU are trainable and not hyperparameters. Certainly, the derivative of the parameters is given by:

(13) $$\frac{\partial h(x_{i}^{r})}{\partial \alpha_{i}}=-1$$

(14) $$\frac{\partial f(x_{i})}{\partial \beta }=-I_{\left\{x_{i}\leq \gamma_{i}\right\}}\cdot (x-\gamma_{i})$$

(15) $$\frac{\partial f(x_{i})}{\partial \gamma_{i}}=-I_{\left\{x_{i}\leq \gamma_{i}\right\}}\cdot \beta_{i}-I_{\left\{x_{i}>\gamma_{i}\right\}}$$

(16) $$\frac{\partial f(x_{i})}{\partial \zeta_{i}}=1$$

where I is an indicator function. For example, for Equation (14), if $x_{i}\leq \gamma_{i}$ is true, the indicator I=1; if not, I=0.

### 3.8. IR-Net

In [20], the authors hold the view that the challenge in the training of high-precision BNNs is mainly due to severe information loss during the training process. Information loss is caused by the approximation between the sign function in forward propagation and the backward gradient. In order to solve the above problem, [20] proposed a new information retention network (IR-Net), which retains the information during the training process and achieves a high-accuracy BNN.

Previously, the vast majority of binarization methods attempted to reduce the quantization error of binarization. However, it is not enough to obtain a good BNN by minimizing the quantization error. Therefore, Libra Parameter Binarization (Libra-PB) in forward propagation was proposed. The key to the Libra-PB design is using both information entropy and quantization error to maximize the information during BNN forward propagation.

Normally, quantization can be defined as:

(17) $$Q_{x}(x)=\alpha B_{x}$$

where α denotes the scaling factor and $B_{x}$ is the binarized input x of the sign function. The optimal quantizer for minimizing the quantization error is given by:

(18) $$\min\;J(Q_{x}(x))=||x-Q_{x}(x)|{|}^{2}$$

According to the definition of information entropy, in BNNs, the entropy of the binary parameter $Q_{x}(x)$ can be calculated by the following formula:

(19) $$H(Q_{x}(x))=H(B_{x})=-p\ln(p)-(1-p)\ln(1-p)$$

where p is the probability of $B_{X}$ taking the value +1. To be more specific, the formula is given by:

(20) $$f(B_{x})=\left\{\begin{array}{ll} p, & if\;B_{x}=+1\\ 1-p, & if\;B_{x}=-1 \end{array}\right.$$

where $B_{X}$ is regarded as a random variable that obeys the Bernoulli distribution. Equation (19) is its probability mass function. Simply pursing the minimization of the quantization error will cause the information entropy of the binarized value to be close to zero in extreme cases. Therefore, Libra-PB takes both the quantization error between non-binarized and binarized values and the information entropy of the binarized value as the optimization objective, defined as:

(21) $$\min\;J(Q_{x}(x))-\lambda H(Q_{x}(x))$$

Under the assumption of a Bernoulli distribution, the information entropy of the quantized values takes the maximum value when p=0.5, which means that the binarized value should have a uniform distribution. Therefore, Libra-PB reshapes the distribution of weight to reduce information loss and quantization error through normalization and balancing operations:

(22) $${\hat{W}}_{std}=\frac{\hat{W}}{\sigma (\hat{W})},\;\hat{W}=W-\bar{W}$$

where σ() is the standard deviation. ${\hat{W}}_{std}$ has two characteristics:

(1) Zero mean to maximize the information entropy of the obtained binarized weights;
(2) Unit norm, which makes the full-precision weights involved in binarization more spread out.

Furthermore, to avoid expensive float-point computation and enhance the representation ability of binarized weights at the same time, the authors also introduced the integer scaling factor S instead of the float-point scaling factor α, such that the calculation of binarization with the scaling factor can be simplified as:

(23) $$Q_{w}({\hat{W}}_{std})=B_{W}<<>>s=sign({\hat{W}}_{std})<<>>S$$

where <<>> denotes a left or right bit-shift operation and S is the number of bits to be shifted. The optimal S can be calculated from the following expressions:

(24) $$\begin{array}{c} {B_{W}}^{*},S^{*}=\mathrm{argmin}||{\hat{W}}_{std}-B_{W}<<>>S|{|}^{2}\;s.t.\;S\in N\\ S^{*}=round(\log_{2}(||{\hat{W}}_{std}|{|}_{1}/n)) \end{array}$$

Thus, finally, Libra Parameter Binarization for forward propagation can be expressed as follows:

(25) $$\begin{array}{c} Q_{w}({\hat{W}}_{std})=B_{W}<<>>s=sign({\hat{W}}_{std})<<>>S\\ Q_{a}(a)=B_{a}=sign(a)\\ Z=(B_{w}⊙B_{a})<<>>S \end{array}$$

The experiments show that the bit-shift operation causes almost no additional inference time or storage consumption.

The authors also proposed the Error Decay Estimator (EDE) to reduce information loss during backward propagation. Due to the discontinuity of binarization, the approximation of the gradient is unavoidable for backward propagation, and this approximation of the sign function brings about two kinds of information loss, including the information loss caused by the decrease in parameter updating ability outside the truncated range and the information loss caused by approximation error within the truncated range. The EDE can preserve the information derived from the loss function in backward propagation through an asymptotic two-stage approximate gradient method.

Firstly, the derivative value of the gradient estimation function is kept close to 1, and then the truncation value is gradually reduced from a large number to 1. Using this rule, the approximation function evolves from close to the identity function to the clip function, which ensures update ability early in the training. Secondly, the truncation is kept at 1, and the derivative curve is gradually evolved to the shape of a step function. Using this rule, the approximation function evolves from a clip function to a sign function, thus ensuring consistency between forward and backward propagation.

### 3.9. AdaBin

AdaBin [17] uses a simple but effective method to adaptively obtain the best binary set—$b_{1},b_{2}(b_{1},b_{2}\in R)$—of weights and inputs for each layer, instead of using a fixed set like  {−1,+1}. This leads to a better fit to different distributions and improves the representation of binarized inputs. Specifically, the authors define a new binary quantization function using the center position and distance of 1-bit numbers. For the weights, the paper proposes an equilibrium approach that aligns the center of the distribution of the binary values with the real-value distribution and then minimizes the KL dispersion. Meanwhile, this paper introduces a gradient-based optimization method to obtain these two parameters for feature binary values and train them in an end-to-end manner.

**Figure 13:**
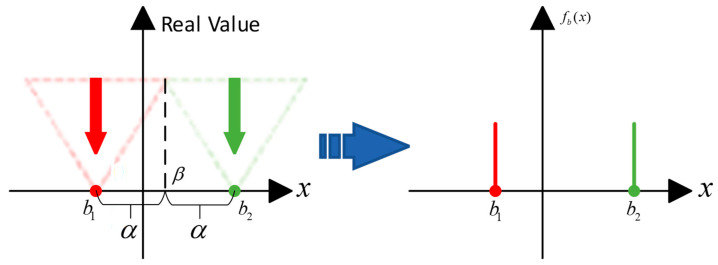
AdaBin quantization flow.

The AdaBin quantizer can adaptively adjust the center position and distance between two clusters, which matches the distributions of binary values and real values well:

(26) $$B(x)=\left\{\begin{array}{ll} b_{1}=\beta -\alpha , & x<\beta \\ b_{2}=\beta +\alpha , & x\geq \beta^{\prime } \end{array}\right.$$

where α is the half distance of the binary values $b_{1}$ and $b_{2}$, and β is the center of the set. B(·) can constrain the binarized values to the set $b_{1},b_{2}$. The set can be rewritten as β−α,β+α. Thus, it is clear that by adjusting α and β, an arbitrary binarized set can be achieved. As shown in Figure 13, given a floating-point distribution of any shape at any position, the position and distance of the binary set will change along with it. The quantization flow is shown as Figure 13. The green part is a set of real numbers that will be quantized as $b_{2}$, while the red part will be quantized as $b_{1}$.

### 3.10. DyBNN

The work of DyBNN [18] is based on ReActNet. ReActNet adopts the static channel-wise thresholds (RSign) on inputs. Rsign is defined as:

(27) $$RSign(x_{i})=\left\{\begin{array}{ll} +1, & x_{i}>\alpha_{i},\\ -1, & x_{i}\leq \alpha_{i} \end{array}\right.$$

DyBNN proposed the dynamic Learning Sign Function (DySign) to modify the input feature map. The authors changed α to a dynamic learnable parameter for each input channel which is fitted by two fully connected layers:

(28) $$\alpha =f(X)=f_{2}(f_{1}(\frac{1}{HW}\sum_{H,W}X))$$

where $f_{1}$ and $f_{2}$ denote two fully connected layers, $f_{1}\in R^{C\times \frac{C}{16}}$ and $f_{2}\in R^{\frac{C}{16}}\times C$. C, H and W are the inputs of channel, height and width, respectively. Finally, DySign can be expressed by:

(29) $$DySign(x_{i})=\left\{\begin{array}{ll} +1, & x_{i}>\alpha_{i},\\ -1, & x_{i}\leq \alpha_{i} \end{array}\right.\alpha_{i}\in \alpha^{1:C}$$

where $\alpha_{i}$ is the threshold value for the i-th channel, which is the i-th output vector of f(X). In experiments, the authors adopted SEblock [63] to learn channel-wise threshold values, $\alpha_{i}$, from the inputs.

### 3.11. Binarized Ghost Module (BGM)

**Figure 14:**
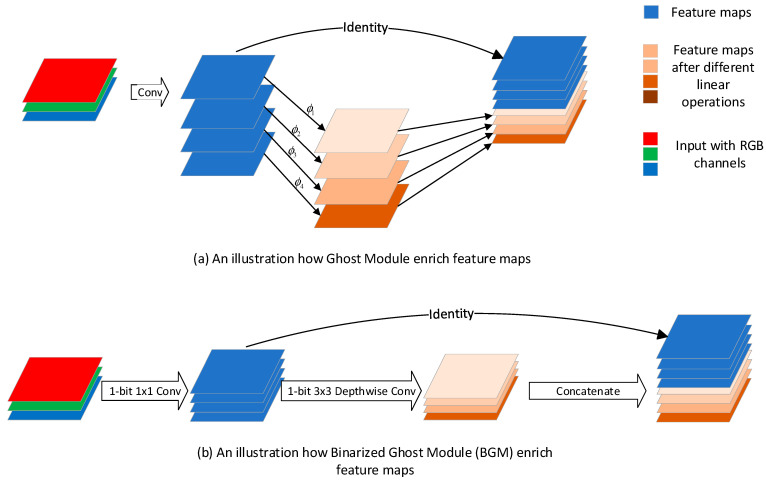
Illustration of Ghost Module at tensor level.

Ruimin Sun et al. also improved the binary version of ReActNet in three different aspects, namely, network structure, loss function and normalization [21]. First of all, in terms of structure, inspired by GhostNet [64], the authors proposed the Binarized Ghost Module (BGM) for saving more information of feature maps with low-complexity computation. As illustrated in Figure 14, different from GhostNet, the BGM does not enrich the information of feature maps by concatenating with feature maps computed by linear operations, but instead boosts the information by the 3 × 3 depth-wise convolutional layer in MobileNet [65]. In Figure 14, ϕ means linear operation.

**Figure 15:**
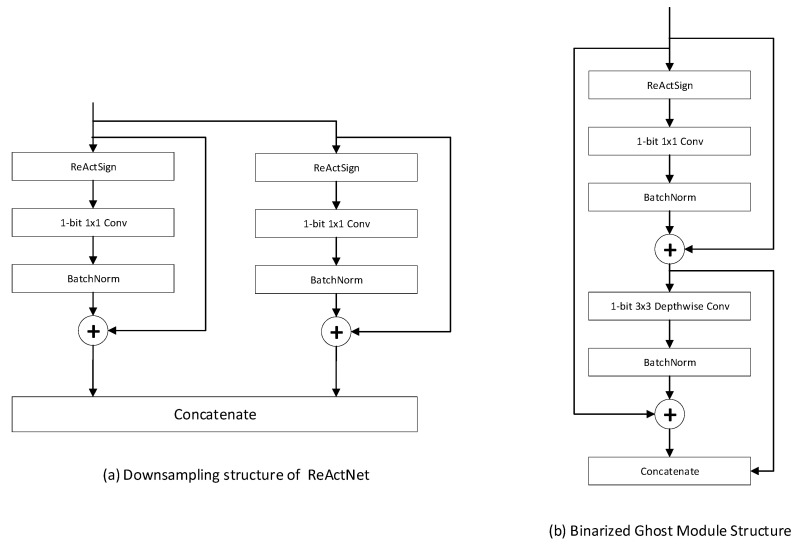
Binarized Ghost Module (BGM) structure.

In detail, the authors still adopted the activation function of ReActNet, called ReActSign, which means that the quantization method in the structure they proposed is the same as the quantization method of ReActNet. The detailed BGM structure is shown in Figure 15.

The authors also found that, in previous works, the last full connection (FC) layer played the role of the classifier and the higher similarity of the distribution of feature maps in the same class could help the classifier to achieve better classification, so they proposed the Label-Aware Loss Function (LLF) to improve performance by enhancing the statistical characteristics of the input of the last FC layer through the L2 norm operation. The LLF can be expressed as:

(30) $$L_{label}=\frac{1}{N}\sum_{i=1}^{N}\frac{1}{N(i)}\sum_{j=1}^{N(i)}||Y_{j}{}^{2}-mean|{|}_{2}^{2}$$

(31) $$mean=\frac{1}{N(i)}\sum_{i=1}^{N(i)}Y_{i}$$

(32) $$L_{total}+=\lambda *L_{label}$$

where Y denotes the feature map of the inputs of the last FC layer, N is the number of categories of the dataset and N(i) indicates the number of feature maps that belong to the i-th category.

They also introduced normalization-based attention [66] to avoid gradient disappearance and accelerate the training process.

### 3.12. IE-Net

Also based on ReActNet, Rui Ding et al. enhanced the information of feature maps by a shared convolutional layer. They designed an information-enhancing module, Information-Enhanced Binary Convolution (IE-BC).

**Figure 16:**
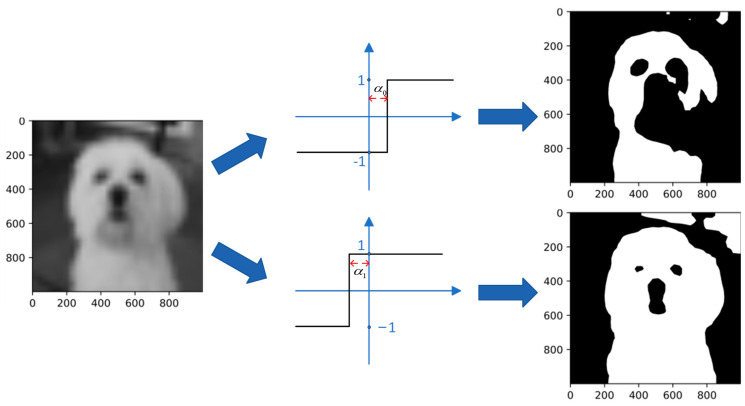
Visualization of influence of activation function distribution.

ReActSign can be regarded as a method to adaptively change the distribution of activation for each channel. Figure 15 indicates the output of the binarized activation function with different channel-wise shifting parameters, and it clearly shows that the ReActSign function may not find an appropriate value which maximizes information retention. Thus, considering the influence of the distribution of the activation function, they adopted multiple ReActSign functions with different shifting parameters. Figure 16 indicates the influence of activation with different distributions.

**Figure 17:**
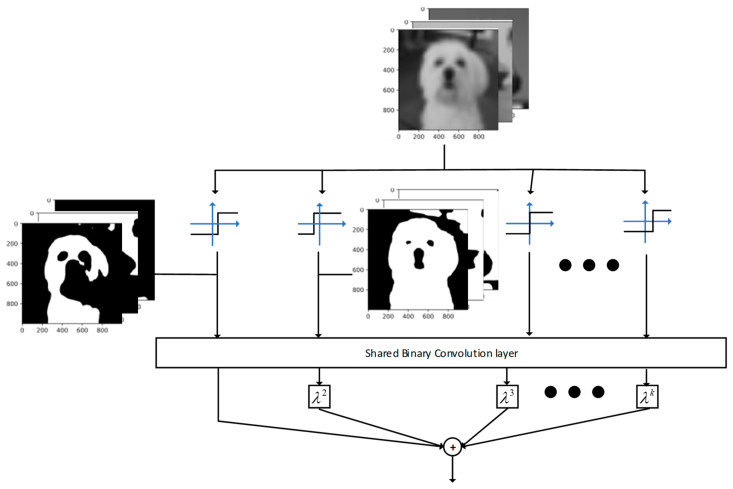
Information-Enhanced Binary Convolution (IE-BC).

Therefore, on the basis of the phenomenon, the authors proposed Information-Enhanced Binary Convolution (IE-BC), which implements multiple paths with activation functions with various channel-wise learnable shifting parameters. The activation function can be formulated as:

(33) $$b_{x}{}^{i,k}=h^{k}(x^{i})=\left\{\begin{array}{ll} +1 & if\;x^{i}\geq \beta^{i,k}\\ -1 & if\;x^{i}<\beta^{i,k} \end{array}\right.$$

where the $h^{k}$ is the k-th Rsign function and $x^{i}$ is the i-th channel of the inputs. For each binarized input, $b_{x}{}^{i,k}$, there is a learnable shifting parameter, $\beta^{i,k}$, for each channel of input. Normally, if K is large, this means that a large number of convolutional layers is needed. At the same time, the memory and computational resource requirements will increase linearly. So, to save memory and computational resources, they decided to use a shared convolutional layer to handle these binarized inputs generated by the multipath RSign function. The expression is given by:

(34) $$\gamma^{k}=(B_{W}⊗B_{x^{k}})\;\alpha$$

where $\gamma_{k}$ is the k-th output and $B_{W}$ and $B_{x^{k}}$ are the binarized convolutional weights and the binarized k-th channel inputs. However, there is only one group of filters contained in a single convolutional layer, which is harmful for the diversity of feature maps. Considering the diversity of feature maps, channel-wise scaling factors, $\lambda^{k}$, were introduced after the convolutional layers to enhance the information of feature maps and compensate for diversity loss. The final output of the convolutional layer can be expressed as follows:

(35) $$\gamma =\gamma^{1}+\sum_{K=2}^{K}\gamma^{k}\lambda^{k}$$

where K is the total number of paths with different parameters. And, for a better understanding, Figure 17 indicates the structure of IE-BC.

To reduce quantization error, taking a cue from IR-Net, the authors also focused on altering the distribution of weight before binarization to maximize the retention of the original information. Distinguished from existing estimators, such as STE [67], the piece-wise polynomial function [14] and EDE [20], they proposed a new adaptive estimator, the Information-Enhanced Estimator (IEE), for training processes. The formula of the IEE is given by:

(36) $$\begin{array}{c} F(x)=\left\{\begin{array}{ll} r(-Sign(x)\frac{3q^{2}x^{2}}{4}+\sqrt{3}qx) & if\;|x|<\frac{2\sqrt{3}}{3q}\\ rSign(x) & otherwise \end{array}\right.\\ q=10^{T_{min}+\frac{e}{E}(T_{max}-T_{min})},\;r=\max(\frac{1}{q},1) \end{array}$$

where $T_{min}=-2,\;T_{max}=1$, and e and E are the current training epoch and the total number of epochs, respectively. In backward propagation, the gradient of the IEE in terms of the input, x, can be calculated by:

(37) $$F’(x)=\left\{\begin{array}{ll} r(\sqrt{3}q+\frac{3q^{2}x}{2}) & if\;-\frac{2\sqrt{3}}{3q}\leq x<0\\ r(\sqrt{3}q-\frac{3q^{2}x}{2}) & if\;0\leq x<\frac{2\sqrt{3}}{3q}\\ 0 & otherwise \end{array}\right.$$

And the gradient of the loss function concerning the weights is given by:

(38) $$\frac{\delta L}{\delta W}=\frac{\delta L}{\delta B_{W}}F’(x)$$

### 3.13. RB-Net

**Figure 18:**
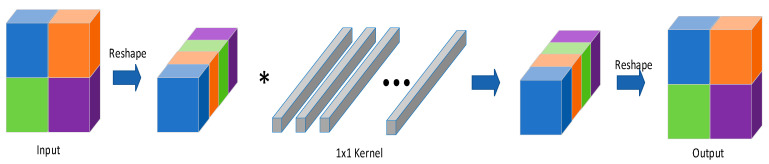
Reshaped Point-Wise Convolution (RPC).

Chunlei Liu et al. further reduced the computational overhead of BNNs with their Reshaped Point-Wise Convolution (RPC) module, which is shown in Figure 18 [22]. Each colour represents a different segmented part. In this case, the input image is segmented into four parts with the same size. And * denotes convolution operation.

The purpose of the module is to replace the large kernel convolutional layer and accelerate computation without loss of information. The speed-up factor is given by:

(39) $$\begin{array}{l} N_{W}=s\times s\\ N_{I}=w_{in}\times h_{in}\\ P=\frac{c\times N_{W}\times N_{I}}{k\times c\times N_{I}}=\frac{N_{W}}{k}=\frac{s^{2}}{k} \end{array}$$

where s is the kernel size of the convolutional layer, $w_{in}$ and $h_{in}$ denote the width and height of the inputs, c represents the number of channels, and k is the factor for reshaping. For example, in Figure 18, k is equal to 4. Finally, the number of channels of convolution after reshaping is k×c. Their experimental results indicate that, for Resnet-18, on ImageNet datasets, their method is nearly 35 times faster compared to the baseline (full precision). And for ResNet-34, there is a 51.55-times speed-up.

**Figure 19:**
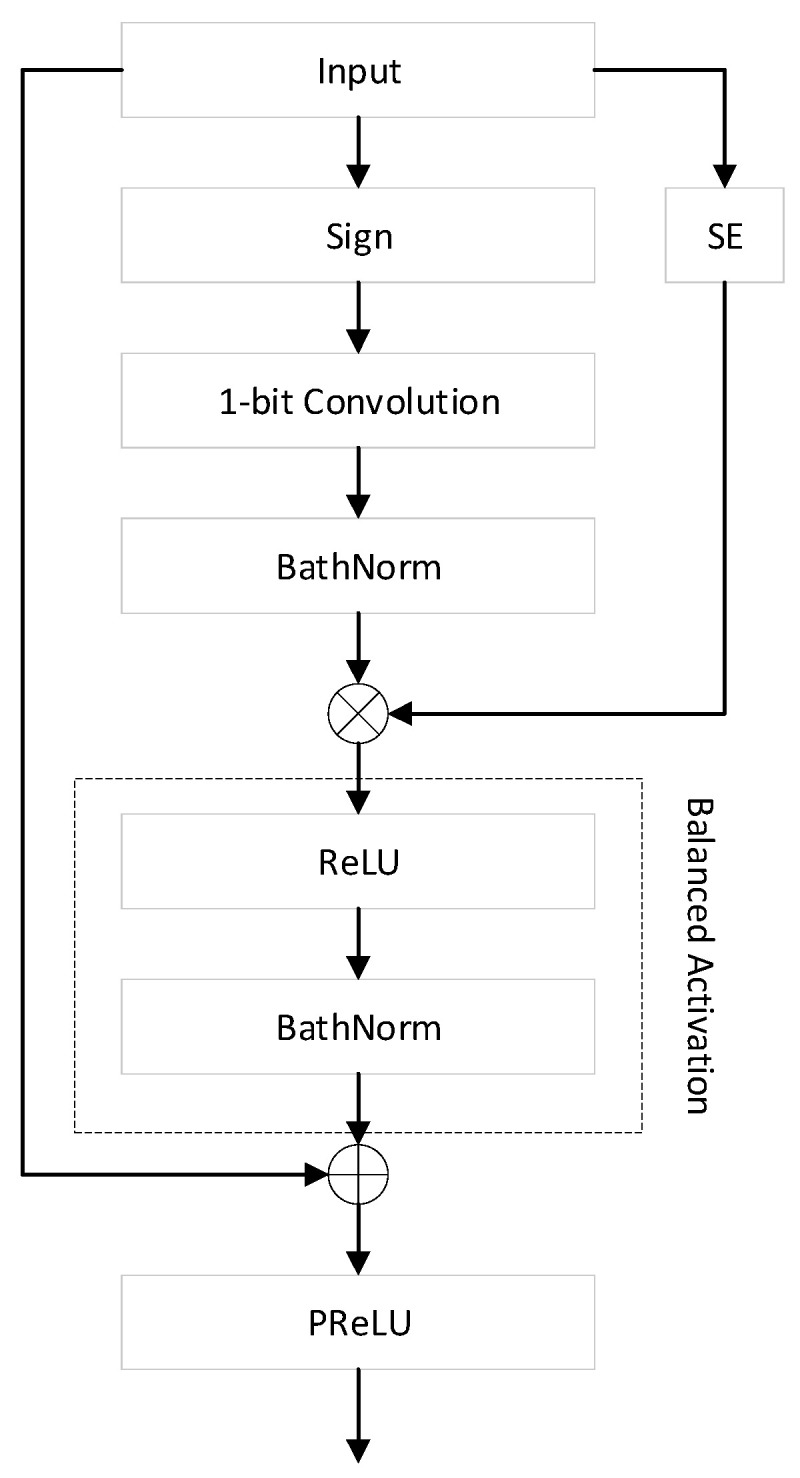
Computation manner of binary convolutional layer with BA.

In terms of binarization, the authors improved both the scaling factor and the distribution, and they were inspired by Real-to-Binary Net [68] and ReActNet, respectively. In Real-to-Binary Net, the scaling factor is calculated in the Squeeze and Excitation (SE) module. Meanwhile, ReActNet improved the performance by altering the distribution of activation. The authors combined these two prior works and proposed the Balanced Activation (BA) module. Figure 19 shows the computation manner of the binary convolutional layer:

## 4. Applications of BNN and FPGA Implementation

The various advantages brought about by BNNs, such as low memory usage, low power consumption and computing acceleration, have garnered significant attention. This section gives some representative works for applications of BNN and FPGA implementation.

In [31], the authors apply binary neural networks (BNNs) to the field of object detection, especially human detection in infrared images. The results show that the performance of their binary neural network is comparable to that of a 32-bit floating-point network while greatly saving computational resources, reducing memory consumption and boosting computational speed by four times. In the field of image super-resolution (ISR), which aims at enhancing the resolution of images in computer vision, BNNs also find utility. Xin et al. [69] proposed a novel model binarization technique called the Bit-Accumulation Mechanism (BAM) to approximate full-precision convolutions. This approach simplifies the heavy computations involved in most ISR systems and improves overall system accuracy.

BiPointNet [38] aims to alleviate the resource constraints of real-time point-cloud applications running on edge devices. The authors of BiPointNet introduced the techniques of Entropy-Maximizing Aggregation (EMA) and Layer-Wise Scale Recovery (LSR) to enhance the performance of binary neural networks (BNNs). Their experiments showed that BiPointNet outperforms other binarization methods significantly, achieving a 14.7-times speed improvement and an 18.9-times saving in terms of storage.

Hirtzlin et al. [70] designed an innovative ultra-low-power neural network hardware circuit system by combining the characteristics of BNNs and resistive memory technologies. They also applied this system to electrocardiogram (ECG) signal tasks. In medical image segmentation, Brahma et al. [71] binarized the network using the distributions of weights and activations. They further improved network accuracy with a non-parametric encoder and decoder. While saving on memory consumption and computational operations, they kept the performance degradation within 3% compared to the full-precision model.

The authors of [72] recognized the significant potential of BNNs in fault diagnosis for edge intelligent power electronics devices. By binarizing a network, they were able to eliminate nearly all floating-point operations, and the model occupied only 7.48 kB of memory without a significant accuracy drop.

Researchers have also explored harnessing the benefits of FPGAs in different applications. Taking into account the power constraints of small IoT devices, the authors of [35] proposed a customized binary precision YOLOv2 model and deployed it on a field-programmable gate array system on chip (FPGA-SoC). The implementation results indicate that their system can achieve low power consumption without sacrificing processing speed. Their system can attain 15.15 frames per second (FPS) and 1.45 W power consumption.

On the other hand, Cladera et al. [73] proposed a hardware-efficient architecture for pedestrian detection with neuromorphic dynamic vision sensors (DVSs) called PPF-BNN, achieved by combining a novel point-process filter (PPF) with a BNN. When deployed on an FPGA and compared to the full-precision architecture (PPF-CNN), PPF-BNN achieved a reduction in latency of approximately 86%. The authors of [74] provided a comparison of the performance of neural networks with different precisions deployed in an FPGA for object-detection applications. This comparison included a full-precision CNN, a quantized convolutional neural network (QCNN) and a BNN.

Frickenstein et al. [37] proposed a binary drivable area detection network (Binary DAD-Net) to reduce model size and accelerate inference. Their model achieved a 14.3-times reduction in computational complexity in an FPGA while requiring only 0.9 MB of memory resources. The authors of [75] proposed a hardware-friendly human activity recognition (HAR) system. This system employs a hardware-friendly pre-processing algorithm and a BNN to classify data from a single three-axis accelerometer. They also validated its low-power characteristics in an FPGA. Huang et al. [26] applied a BNN in the field of agriculture. They utilized the BNN for detecting pest and disease severity in crops and deployed it in their FPGA experimental platform. Their results showed that their system consumed less than 17% of the resources of the FPGA.

## 5. Comparison of BNN Architectures for FPGA Implementation

This section provides comparisons of BNN architectures and two tools for FPGA implementation on established datasets. We used the FINN and HLS4ML tool flow for the hardware implementation.

In our experiments, we used the sign function for the binarization of weights and activations, and STE to train the network in the FINN tool flow, since FINN still only supports the simplest BNNs and does not support any kind of scaling factor. HLS4ML can support two types of quantization methods, the statistical scaling factor of [12] and the shift integer scaling factor of [20].

The implementation of BNNs is based on two boards: Z7P and PYNQ-Z2. Z7P is a development board that uses the same family of Xilinx parts (xczu7ev-ffvc1156-2-i) as ZCU104. The Xilinx part of PYNQ-Z2 is XC7Z020CLG400-1. The hardware resources are listed below.

It is clear from Table 2 that Z7P has more on-chip resources than PYNQ-Z2, which means it can afford networks with deeper structures and more parameters.

### 5.1. MNIST

MNIST is a dataset of handwritten digits from the National Institute of Standards and Technology (NIST) [24]. The training set consists of handwritten digits from 250 different people, 50% of whom are high school students and 50% of whom are staff members of the Census Bureau. Similarly, the test set also comprises the same percentage of handwritten digits.

CNV-4 is a convolutional model consisting of two convolutional layers and two fully connected layers, with each convolutional layer paired with batch normalization, a sign activation function and a max-pooling layer. MLP-4 is formed by four fully connected layers with batch normalization and the sign activation function. Normally, when the bit-width of the two values is relatively small (e.g., less than 10 bits), we can use a flip-flop (FF) in the FPGA to calculate the multiplication. Based on the above discussion, if the weights and activations in the model have been binarized to 1 and −1, the FF is capable of undertaking multiplication operations on them. Meanwhile, as we discussed in Section 2.5.2, FINN converts the batch normalization (BN) and the activation function into a single threshold operation and proposes MTVU to optimize and accelerate the matrix and vector multiplication. Therefore, there is no DSP usage in the resource consumption of all the BNN models in our experimental results. The FINN experimental results on the MNIST dataset are shown in Table 3.

For the baseline model, all operations are still floating point, which inevitably results in ultra-high utilization of DSPs and even makes deployment in FPGAs impossible. DSP usage is highly dependent on precision. This is the reason why the utilization of DSPs will change abruptly between the baseline and three BNNs. From Table 4, we can see that the three quantization methods improve the accuracy to a certain extent and do not result in excessive costs in terms of computational resources.

Unlike FINN, HLS4ML focuses on a more efficient and flexible way of controlling DSPs to handle matrix or vector multiplication operations with different levels of precision. As discussed in Section 2.2, the reuse factor is an important parameter for parallelism which determines whether a BNN architecture saves resource consumption by reusing the same set of DSPs or uses multiple DSPs to compute multiplication operations in parallel. In other words, the reuse factor indicates the number of times each group of DSPs will be reused. And, as our results show, as the reuse factor increases, the usage of DSPs must decrease. However, the price is a degradation in parallelism, which is also directly reflected in latency. Latency means how long it takes the platform to complete the computation in the network.

### 5.2. CIFAR10

The CIFAR10 [76] dataset consists of 60,000 three-channel images in 10 categories, including 6 different animal categories and 4 different vehicle categories. Each category contains 6000 images, and each image has 32 × 32 pixels. The 60,000 images are separated into a 50,000-image training set and a 10,000-image test set.

The architecture of CNV-8 is based on VGG-small, consisting of five convolutional layers with 128, 128, 256, 256, and 512 channels, respectively, and two fully connected layers. Due to the size of the BRAM, the implementation of CNV-8 on Z7P and PYNQ-Z2 differs in the number of channels for each convolutional layer. PYNQ-Z2 can only afford CNV-8 with half the number of channels due to BRAM limitations. As shown in Table 5, reducing the number of channels significantly decreases BRAM utilization because fewer weights need to be stored. The inference time for CNV-8 with half the number of channels for one picture is over two times faster than for CNV-8.

Furthermore, PYNQ-Z2 is the officially supported board for FINN, so it benefits from special resource optimizations, including memory and protocol control. Therefore, in this experiment, PYNQ-Z2 outperformed Z7P in terms of inference speed for the same BNN structure. However, theoretically, Z7P is a more powerful board than PYNQ-Z2, given its more extensive hardware resources, as evident in Table 2. The advantages of having more extensive hardware resources were also demonstrated in this experiment, particularly with respect to Z7P’s ability to support a larger network (CNV-8, without changing the number of channels) without high resource utilization, making it feasible for running large networks on the Z7P and enabling exploratory possibilities.

However, this enhanced capability is a double-edged sword, as seen in all the experimental results. Z7P’s energy consumption is twice or more than that of the PYNQ-Z2, which may be unfavorable for certain applications that require devices to operate with ultra-low energy consumption.

## 6. Challenges and Future Directions

This section summarizes the current challenges and potential future research directions for BNNs from prior works. According to the challenges, the directions can be separated into: (1) online training, (2) various applications and (3) generalizability.

### 6.1. Online Training

Most of the studies on BNNs are based on offline training, and training will take a long time. Online training will be an efficient way of training a model rapidly. In [77], the authors proposed a solution for online training on a macrochip with limited resources. They used the binary value for inferences and less than 8-bit values for training. The results showed that the accuracy on MNIST was close to 97% in a CMOS process with 16 Mb RRAM. The work demonstrates the potential of online training. In a newer work [78], the authors proposed a new continual learning solution and conducted experiments and analyzed the effectiveness and feasibility of a BNN in on-chip training.

### 6.2. Various Applications

Binary neural networks represent parameters and activations using {1,−1} or bipolar values, which result in lower computational and storage requirements. However, they also suffer from performance degradation. To minimize the error between the full-precision model and the binary model, our survey has summarized various techniques. While most studies still focus on image classification, there are only a few applications in other areas. Several application domains, such as security and point-cloud tasks, aim to achieve low latency and efficient resource utilization through the properties of BNNs.

### 6.3. Generalizability

One of the important factors to consider is which architecture is the best choice for binarization. It remains unclear what kind of architecture can effectively preserve information during binarization. Despite the existence of various architectures proposed in prior works that have demonstrated impressive results, these architectures are often tailored to specific tasks. The lack of a general network topology is an issue that needs to be urgently addressed.

## 7. Conclusions

This paper has provided a comprehensive review of BNNs for implementation in FPGA hardware. The survey covered different aspects, such as BNN architectures and variants, design and tool flows for FPGAs, and various applications for BNNs. In the current times, even though BNNs significantly compress models and speed up inference compared to traditional DNNs, they cannot achieve the same level of accuracy as their full-precision counterparts. However, some improvements have been made to narrow this accuracy gap. By adding gain terms, incorporating learnable gain terms, modifying activation functions and enhancing network architecture, among other techniques, BNNs have managed to achieve relatively high accuracy while still maintaining high-speed execution and small model size. Several challenges remain to be resolved, such as online training for BNNs, the customizability of BNNs for targeted applications to avoid performance degradation and the generalization of network topologies for BNNs. With further research into BNN implementation in FPGAs and ASICs, several tool flows, such as FINN and HLS4ML, have emerged, simplifying the process of implementing BNNs and QNNs and making them more accessible for designers.

## Figures and Tables

**Table 1:** BNN performance comparisons for ImageNet.

Method	Architecture	Top-1 Accuracy (%)	Top-5 Accuracy (%)	$\mathrm{BOPs}\;(\times 10^{9}$)	$\mathrm{FLOPs}\;(\times 10^{8}$)	$\mathrm{OPs}\;(\times 10^{8}$)
BWM [12]	AlexNet	56.8	79.4	1.70	1.20	1.47
	ResNet-18 [59]	60.8	83.0	-	-	-
	GoogLeNet [60]	65.5	86.1	-	-	-
XNOR [12]	AlexNet	44.2	69.2	-	-	-
	ResNet-18	51.2	73.2	1.70	1.33	1.60
Bi-Real-Net [14]	ResNet-18	56.4	79.5	1.68	1.39	1.63
	ResNet-34	69.2	83.9	3.53	1.39	1.93
XNOR++ [61]	ResNet-18 (α,β,γ)	57.1	79.9	1.695	1.33	1.60
BinaryDenseNet [15]	BinaryDenseNet28	60.7	82.4	-	-	2.58
	BinaryDenseNet37	62.5	83.9	-	-	2.71
	BinaryDenseNet37-dilated	63.7	84.7	-	-	2.20
MeliusNet [62]	MeliusnetC	64.1	-	5.47	1.29	2.14
	Meliusnet42	69.2	-	9.69	1.74	3.25
	Meliusnet59	71.0	-	18.3	2.45	5.32
ReActNet [16]	ReActNet-A(based on MobileNet-v1 [63])	69.4	-	4.82	0.12	0.87
	ReActNet-B(based on MobileNet-v1)	70.1	-	4.69	0.44	1.63
	ReActNet-C(based on MobileNet-v1)	71.4	-	4.69	1.40	2.14
IR-Net	ResNet-18	58.1	80.0	1.68	1.40	1.67
	ResNet-34	62.9	84.1s		1.93	
AdaBin [17]	AlexNet	53.9	77.6	-	-	-
	ResNet-18	63.1	84.3	1.69	1.410	1.67
	ReActNet	66.4	86.5	-	-	-
	ResNet-34	66.4	86.6	-	-	-
DyBNN [18]	ResNet-18	67.4	87.4	-	-	-
	MobileNet-v1	71.2	89.8	-	-	-
BGM [21]	ReActNet-B(based on MobileNet-v1)	71.4	-	-	-	-
IE-Net	ResNet-18	61.4	83.0	-	1.63	-
	ResNet-34	64.6	85.2	-	1.93	-
RB-Net	ResNet-18	66.8	87.1	-	0.52	-
	ResNet-34	70.2	89.2	-	0.71	-

**Table 2:** On-chip resources.

	PYNQ-Z2	Z7P
	(XC7Z020CLG400-1)	(XCZU7EV-2FFVC1156-MPSoC)
System Logic Units	13.3 K	504 K
DSPs	220	1728
LUTs	5.3 K	230.4 K
LUTRAM	1.74 K	101.76 K
FF	10.64 K	460.8 K
Block RAM (BRAM)	140	312

**Table 3:** BNN performances and hardware costs using FINN on MNIST.

Model	Board	Quantization Method	Accuracy	LUTs (Utilization)	LUTRAM	FF	BRAM	On-Chip Power (W)
MLP-4	Z7P	BNN (non-scaling)	88% ± 1%	14,222 (6%)	1707 (1.6%)	22,853 (5%)	13.5 (4%)	3.556
MLP-4	PYNQ-Z2	BNN (non-scaling)	88% ± 1%	11,579 (22%)	1197 (6%)	17,981 (16%)	14.5 (10%)	1.598
CNV-4	Z7P	BNN (non-scaling)	92% ± 1%	21,417 (9%)	3734 (4%)	29,899 (7%)	14 (5%)	3.721
CNV-4	PYNQ-Z2	BNN (non-scaling)	92% ± 1%	18,773 (35%)	2198 (12%)	24,925 (23%)	42(30%)	1.808

**Table 4:** BNN performances and hardware costs using HLS4ML.

Model	Board	Quantization Method	Accuracy	BRAM-18K (Utilization)	DSP48E	FF	LUT	Reuse Factor	Latency (ms) [min, max]
CNV-4	Z7P	Baseline (non-binarized)	98% ± 1%	83 (13%)	3734 (216%)	57,520 (12%)	178,939 (77%)	128	-
CNV-4	Z7P	BNN (non-scaling)	76% ± 1%	103 (16%)	81 (4%)	41,158 (8%)	61,108 (26%)	64	[0.217, 0.219]
CNV-4	Z7P	XNOR-Net	82% ± 1%	103 (16%)	81 (4%)	41,047 (8%)	61,172 (26%)	64	[0.217, 0.219]
CNV-4	Z7P	XNOR-Net (integer shifting scaling factor)	83% ± 1%	296(16%)	502 (4%)	58,587 (8%)	83,152 (26%)	8	[0.164, 0.166]
CNV-4	Z7P	XNOR-Net (integer shifting scaling factor)	83% ± 1%	181 (29%)	251 (14%)	46,028 (9%)	72,021 (31%)	16	[0.170, 0.172]
CNV-4	Z7P	XNOR-Net (integer shifting scaling factor)	83% ± 1%	139 (22%)	161 (9%)	43,170 (9%)	64,916 (28%)	32	[0.186, 0.188]
CNV-4	Z7P	XNOR-Net (integer shifting scaling factor)	83% ± 1%	103 (16%)	81 (4%)	41,045 (8%)	61,048 (26%)	64	[0.217, 0.219]

**Table 5:** BNN performances and hardware costs using FINN on Cifar10.

Model	Board (Tool)	Quantization Method	Accuracy	LUTs (Utilization)	LUT RAM	FF	BRAM	On-Chip Power (W)	Time (s/Picture)
CNV-8 VGG-small	Z7P	BNN	78% ± 2%	41,713 (21%)	3755 (4%)	53,280 (12%)	194 (62%)	4.473	0.35
CNV-8 VGG-small (half the number of channels)	Z7P	BNN	75% ± 2%	27,179 (12%)	2653 (3%)	32,359 (7%)	77 (25%)	3.901	0.16
CNV-8 VGG-small (half the number of channels)	PYNQ-Z2	BNN	75% ± 2%	25,318 (48%)	2285 (13%)	31,608 (29%)	90 (64%)	1.955	0.05
